# Smoking, *Porphyromonas gingivalis* and the immune response to citrullinated autoantigens before the clinical onset of rheumatoid arthritis in a Southern European nested case–control study

**DOI:** 10.1186/s12891-015-0792-y

**Published:** 2015-11-04

**Authors:** Benjamin A. Fisher, Alison J. Cartwright, Anne-Marie Quirke, Paola de Pablo, Dora Romaguera, Salvatore Panico, Amalia Mattiello, Diana Gavrila, Carmen Navarro, Carlotta Sacerdote, Paolo Vineis, Rosario Tumino, David F. Lappin, Danae Apazidou, Shauna Culshaw, Jan Potempa, Dominique S. Michaud, Elio Riboli, Patrick J. Venables

**Affiliations:** Rheumatology Research Group, Centre for Translational Inflammation Research, Queen Elizabeth Hospital Birmingham, Birmingham, B15 2WB UK; Kennedy Institute of Rheumatology, University of Oxford, Oxford, UK; School of Public Health, Imperial College London, London, UK; CIBER-OBN (Fisiopatología de la Obesidad y Nutrición), Madrid, Spain; Department of Clinical and Experimental Medicine, Federico II University of Naples, Naples, Italy; Department of Epidemiology, Murcia Regional Health Council, Murcia, Spain; CIBER Epidemiología y Salud Pública (CIBERESP), Murcia, Spain; Human Genetics Foundation, Turin, Italy; Cancer Registry and Histopathology Unit, “Civic - M.P.Arezzo” Hospital, ASP Ragusa, Ragusa, Italy; University of Glasgow Dental School, University of Glasgow, Glasgow, UK; Faculty of Biochemistry, Biophysics and Biotechnology, Jagiellonian University, Krakow, Poland; Oral Health and Systemic Research Group, School of Dentistry, University of Louisville, Louisville, USA; Department of Epidemiology, Brown University School of Public Health, Providence, USA

**Keywords:** Smoking, Rheumatoid arthritis (RA), Anti-citrullinated protein antibodies (ACPA), *Porphyromonas gingivalis*, Citrullination

## Abstract

**Background:**

Antibodies to citrullinated proteins (ACPA) occur years before RA diagnosis. *Porphyromonas gingivalis* expresses its own peptidylarginine deiminase (PPAD), and is a proposed aetiological factor for the ACPA response. Smoking is a risk factor for both ACPA-positive RA and periodontitis. We aimed to study the relation of these factors to the risk of RA in a prospective cohort.

**Methods:**

We performed a nested case–control study by identifying pre-RA cases in four populations from the European Prospective Investigation into Cancer and nutrition, matched with three controls. Data on smoking and other covariates were obtained from baseline questionnaires. Antibodies to CCP2 and citrullinated peptides from α-enolase, fibrinogen, vimentin and PPAD were measured. Antibodies to arginine gingipain (RgpB) were used as a marker for *P.gingivalis* infection and validated in a separate cohort of healthy controls and subjects with periodontitis.

**Results:**

We studied 103 pre-RA cases. RA development was associated with several ACPA specificities, but not with antibodies to citrullinated PPAD peptides. Antibody levels to RgpB and PPAD peptides were higher in smokers but were not associated with risk of RA or with pre-RA autoimmunity. Former but not current smoking was associated with antibodies to α-enolase (OR 4.06; 95 % CI 1.02, 16.2 versus 0.54; 0.09-3.73) and fibrinogen peptides (OR 4.24; 95 % CI 1.2-14.96 versus 0.58; 0.13-2.70), and later development of RA (OR 2.48; 95 % CI 1.27-4.84 versus 1.57; 0.85-2.93), independent of smoking intensity.

**Conclusions:**

Smoking remains a risk factor for RA well before the clinical onset of disease. In this cohort, *P.gingivalis* is not associated with pre-RA autoimmunity or risk of RA in an early phase before disease-onset. Antibodies to PPAD peptides are not an early feature of ACPA ontogeny.

## Background

Rheumatoid arthritis (RA) is a chronic inflammatory disease of synovial joints with a prevalence of 0.5-1 %. An autoimmune pathogenesis is supported by the strong association with anti-citrullinated protein/peptide antibodies (ACPA), which occur in 50-80 % of cases and may be detected many years before the onset of clinical symptoms. There may therefore be a lengthy pre-clinical stage of RA with different phases, including systemic autoimmunity without symptoms, symptoms in the absence of arthritis, and unclassified arthritis [1]. Importantly, risk factors for one phase may not be so for others. ACPA responses are characterised by differing but overlapping patterns of reactivity to several citrullinated autoantigens, and the number of such specificities increase prior to the clinical onset of joint disease [2–4]. Recent data suggest that the epitopes that break tolerance may differ between individuals, and from those most strongly associated with the clinical onset of disease, indicating that epitope spreading may be intimately related to disease onset [5].

The presence of ACPA in the absence of clinical or imaging evidence of synovitis suggests an origin outside of the joint, and commonly proposed sites include periodontal tissue [6] and the lungs [7]. Along with others, our group has investigated the relationship of periodontitis with RA, and infection with *Porphyromonas gingivalis* in particular [8–12]. *P.gingivalis* codes for a bacterial peptidyl arginine deiminase (PPAD) enzyme that differs from human PAD enzymes but is capable of citrullinating human proteins [11, 13]. We have also shown that citrullinated peptides from PPAD are a target of the ACPA response in a subset of patients with RA [10], though it remains unclear whether this response is driven by citrullination of PPAD [14] or whether it is part of a polyreactive ACPA response.

Smoking is a known risk factor for the development of ACPA-positive RA in Northern European and American populations [15–17], but few previous studies have addressed the relationship of smoking with specific subsets of ACPA in the years before disease onset [18], or whether smoking is a risk factor for RA in other populations. Smoking is also a risk factor for periodontitis, [19, 20] and so an untested hypothesis could be that smoking increases the risk of RA through promoting periodontal disease.

In this study we present a cohort of southern European subjects who donated blood prior to the onset of RA, and investigate the associations of smoking, antibodies to *P. gingivalis* arginine gingipain (RgpB), and citrullinated PPAD peptides, with the risk of RA and pre-RA autoimmunity.

## Methods

### Cohorts

EPIC is a multicentre, pan-European prospective cohort study designed to investigate the association between diet and cancer, as well as other diseases, in apparently healthy populations [21]. We undertook a nested case–control study to investigate risk factors for RA, by identifying incident RA cases and matched controls amongst subjects enrolled in four EPIC cohorts: Naples (5062 females, recruited 1993–97), Turin (6047 males and 4557 females; recruited 1993–98) and Ragusa (3053 males and 3350 females; recruited 1993–97) in Italy, and Murcia (2685 males and 5831 females, recruited 1992–96) in Spain.

Baseline questionnaires collected detailed data on diet, physical activity and lifestyle factors (current and lifetime history). Smoking data included the age at starting and stopping, and the number of cigarettes smoked currently and at ages <30, 30–40, 40–50 and >50. This allowed calculation of a lifetime average number of cigarettes/day. Alcohol intake at recruitment was estimated by grams per day. Physical examination collected data on height, weight and waist circumference. In each centre, blood for serum was collected at baseline, transferred to a local laboratory at 5-10 °C whilst protected from light, and following processing, stored in 0.5 ml straws at −196 °C in liquid nitrogen. Samples for this study were retrieved and sent on dry ice to a central laboratory where they were analysed blinded to case/control status.

### Case ascertainment

In Murcia, RA cases were identified by linkage with primary health care records (International Classification of Primary Care code L88) and prescriptions of disease-modifying anti-rheumatic drugs, and linkage using ICD codes with hospital discharge (ICD9 – 714) and mortality databases (ICD10 – M05 and M06). In Naples, RA cases were identified by linkage with hospital discharge databases and information from systematic telephone follow-up of participants. In Turin, RA cases were identified by linkage with hospital discharge databases and a drug prescription database with a disease-specific code. In Ragusa, cases were identified by linkage with hospital discharge databases. All RA case identification was undertaken in 2011.

RA cases were then validated by medical record review to confirm a physician diagnosis of RA and to confirm date of diagnosis. Additional information was extracted to assess fulfilment of 1987 (and 2010) ACR classification criteria [22, 23].

In Murcia, 80 potential cases were identified and 38 validated as having a physician’s diagnosis of RA, all of which met 1987 criteria. In Naples, 24 of 48 potential cases were validated, all of which met 1987 criteria. In Turin, 54 of 59 potential cases were validated however, due to missing data, only 20 were confirmed as meeting 1987 criteria, with an additional case meeting 2010 criteria. In Ragusa 8 of 9 cases were confirmed as having a physician diagnosis of RA, but again, due to missing data, only 1 case could be confirmed as meeting 2010 criteria. This yielded a total of 124 cases with a physician diagnosis of RA (84 of which were also confirmed as meeting classification criteria), of which 113 were incident cases (pre-RA), having being diagnosed with RA after their baseline visit, 10 prevalent, with a diagnosis preceding their baseline visit, and one had a missing diagnosis date. Samples were available for 103 pre-RA cases with a physician diagnosis, used in the analysis here, of which 62 (60 %) were also confirmed as meeting 1987/2010 criteria, the remainder having incomplete available data to ascertain fulfilment of criteria. However where full data was available, there was a high concordance between physician diagnosis and meeting classification criteria. Mean age at enrolment was 51 (sd 7.5) and 78 % were female. The median time to diagnosis in the pre-RA cases was 7 years (range 1.7-15.8 years).

Three controls were randomly selected from living cohort members and matched for every individual case by age at blood collection (±1 year), sex, centre, date (±2 months) and time (±3 h) of baseline blood collection, and fasting status at blood collection (<3/3-6/>6 h).

Consent was given by all participants and the study was approved by the International Agency for Research on Cancer (IARC) review board as well as by the local committees of participating EPIC centres.

### Laboratory methods

IgM rheumatoid factor (RF) was assayed by particle agglutination following the manufacturer’s recommendations (Serodia-RA, Fujirebio Inc, Tokyo) and with agglutination at a dilution of 1:40 considered positive.

### ELISA method for peptides

Anti-CCP2 antibodies were measured by commercial ELISA (DIASTAT, Eurodiagnostica, Malmö, Sweden). Sera were further tested for antibodies to immunodominant peptides from 3 established citrullinated autoantigens in RA, α-enolase (CEP-1; ^5^KIHA-cit-EIFDS-cit-GNPTVE^21^) [9], vimentin (cVim; ^59^VYAT-cit-SSAV-cit-L-cit-SSVP^74^) and fibrinogen (cFib; β chain ^36^NEEGFFSA-cit-GHRPLDKK^52^) [24], as well as two citrullinated PPAD peptides (CPP3, AKTDSYWT-cit-DYTGWFAMYD and CPP5, LAPNKILI-cit-KVPDNHPQH) [10], with cysteines at the end of each peptide to facilitate cyclisation. Arginine-containing control peptides for all assays were run in parallel. Ninety-six well plates were coated with peptide at 10 μg/ml overnight at 4 °C, washed with PBS-Tween (0.05 %) and blocked with 2 % BSA for 2 h. Samples were diluted 1:100 in RIA buffer (10 mM Tris, 1 % BSA, 350 mM NaCl, 1 % Triton-X, 0.5 % Na-deoxycholate, 0.1 % SDS) and added in duplicate for 1.5 h. Plates were washed as described above and incubated with peroxidase-conjugated mouse anti-human IgG (Hybridoma Reagent Laboratory, Baltimore, USA) (diluted 1:3000) for 1 h at room temperature. After a final wash, bound antibodies were detected with tetramethylbenzidine (TMB) substrate (Biolegend, San Diego, USA). The reaction was stopped by the addition of 1 M H_2_SO_4_ and absorbance measured at 450 nm. A standard curve was used for the ELISAs except those incorporating uncitrullinated fibrinogen and α-enolase peptides where reactivity was rare.

### ELISA method for RgpB

ELISA plates were coated with 100 μl/well of 5 μg/ml RgpB or coating buffer alone (50 mM carbonate buffer, pH 9.5), and incubated overnight at 4 °C. Wells were washed with PBS-Tween (0.05 %) and blocked with 2 % BSA in PBS for 2 h at room temperature. Serum diluted 1:200 in RIA buffer, was added in duplicate, and incubated for 1.5 h at room temperature. Plates were washed as described above and incubated with peroxidase-conjugated mouse anti-human IgG (1:3000) in RIA-buffer for 1 h. After a final wash, bound antibodies were detected with TMB substrate. The reaction was stopped after 5 min by the addition of 1 M H_2_SO_4_ and absorbance measured at 450 nm. A standard curve was included on each plate.

### Validation of anti-RgpB antibodies as a marker of *P.gingivalis* infection

39 patients with previously untreated chronic periodontitis were recruited from Glasgow Dental Hospital, along with 30 healthy control subjects of a similar age and gender, as previously described [25, 26]. Each periodontitis subject had a minimum of 18 teeth with at least two non-adjacent teeth with periodontal pockets of ≥5 mm, 5 mm or more of attachment loss, and radiographic evidence of bone loss. Blood and dental plaque samples were taken prior to treatment, and carriage of *P.gingivalis* was determined by PCR of bacterial plaque DNA using *P.gingivalis*-specific 16S primers as previously described [25, 26]. Serum samples for these subjects were tested for anti-RgpB antibody levels.

### Statistical analysis

The 98^th^ percentile of the non-RA control population included in this study was used to calculate cut-offs for positivity for the ACPA and RgpB ELISAs, with the exception of the commercial anti-CCP2 assay where the manufacturer’s cut-off (>5 units) was used. We also categorised the anti-RgpB antibody response into four groups based on antibody levels.

Differences in median antibody levels among cases and controls were compared using the Mann–Whitney U Test. Differences in antibody frequencies among cases and controls were tested with the Chi square test. Odds ratios with 95 % confidence intervals were calculated using conditional logistic regression models. Models were further adjusted for body-mass index (BMI), waist circumference, smoking, education, physical activity, and alcohol intake. These covariates were obtained from baseline questionnaires at enrolment. Models of smoking and ACPA were only adjusted for age and sex, due to the smaller sample size of these groups.

## Results

### Association of ACPA with pre-RA

The frequency of positive antibodies in the pre-RA cases compared with controls were: anti-CCP2 (23 % vs 3 %; *p* < 0.001), anti-cFIB (18 % vs 2 %; *p* < 0.001), anti-CEP-1 (15 % vs 2 %; *p* < 0.001), and anti-cVIM (6 % vs 2 %; *p* < 0.006). IgM RF was present in 22 % of cases and 4 % of controls (*p* < 0.001). The highest adjusted OR for development of RA was seen for anti-cFib antibodies (OR 10.4; 95 % CI 3.4–32.1; Table 1). Similar, statistically significant ORs were also observed with anti-CCP2 and anti-CEP-1 antibodies, and a trend seen with anti-cVim antibodies.

**Table 1:** Odds ratios for the association of antibodies to citrullinated peptides, their uncitrullinated controls, rheumatoid factor (RF) and RgpB, with pre-RA (*n* = 103). Conditional logistic regression was used with matched controls are referent (*n* = 309). Cut-off for antibody positivity is the 98^th^ percentile of matched controls with the exception of anti-CCP2 and RF. RF; rheumatoid factor. CEP1, cFib and cVim; citrullinated peptides from α-enolase, fibrinogen and vimentin respectively. REP1, Fib and Vim are the arginine-containing control peptides. CPP3 and CPP5 are immunodominant peptides from PPAD, and RPP3 and RPP5 are the arginine-containing control peptides. RgpB; arginine gingipain

Antibodies	Controls *n* = 309	Pre-RA *n* = 103	Crude		Fully adjusted model	
	n (%)	n (%)	OR (95 % CI)	*p*-value	OR (95 % CI)^a^	*p*-value
CCP2	10 (3)	24 (23)	9.94 (4.28–23.07)	<0.001	9.46 (3.89–23.00)	<0.001
RF	12 (4)	23 (22)	5.73 (2.79–11.76)	<0.001	5.84 (2.66–12.86)	<0.001
CEP1	6 (2)	15 (15)	7.11 (2.75–18.35)	<0.001	7.26 (2.53–20.82)	<0.001
REP1	6 (2)	2 (2)	0.77 (0.15–3.93)	0.748	1.17 (0.22–6.30)	0.853
cFIB	5 (2)	8 (18)	12.01 (4.06–35.55)	<0.001	10.44 (3.39–32.10)	<0.001
FIB	5 (2)	2 (2)	1.00 (0.19–5.19)	1.000	1.05 (0.20–0.57)	0.957
cVIM	3 (2)	5 (6)	2.84 (0.66–12.05)	0.157	4.84 (0.94–24.94)	0.059
VIM	5 (2)	4 (4)	2.00 (0.53–7.50)	0.304	2.47 (0.62–9.76)	0.199
CPP3	6 (2)	2 (2)	1.00 (0.20–4.95)	1.000	1.54 (0.28–8.31)	0.618
RPP3	5 (2)	5 (5)	2.88 (0.83–9.97)	0.095	3.76 (0.89–15.86)	0.071
CPP5	6 (2)	5 (5)	2.41 (0.73–7.91)	0.147	2.47 (0.71–8.54)	0.154
RPP5	5 (2)	2 (2)	1.20 (0.23–6.19)	0.827	1.78 (0.31–10.39)	0.521
RgpB	12 (4)	3 (3)	0.66 (0.17–2.62)	0.555	0.60 (0.15–2.46)	0.479
RGPB categories						
1 (<1280)	123 (44)	51 (50)	ref	-	ref	-
2 (≥1280)	75 (27)	29 (28)	0.93 (0.54–1.60)	0.787	0.98 (0.55–1.77)	0.957
3 (≥2560)	68 (24)	20 (19)	0.74 (0.39–1.38)	0.341	0.85 (0.44–1.68)	0.647
4 (≥5120)	12 (4)	3 (3)	0.59 (0.14–2.45)	0.472	0.57 (0.13–2.44)	0.449

^a^ Adjusted for BMI, waist circumference, smoking, education, physical activity, and alcohol intake

Time from blood sampling to diagnosis was dichotomised at the median and the frequency of positivity for antibodies in pre-RA samples ≥7 years before diagnosis versus <7 years is shown in Table 2, indicating that all of the antibodies increase in frequency closer to diagnosis and that no specificity amongst those analysed was particularly prominent in very early pre-diagnosis autoimmunity. Median antibody levels to CEP-1 were higher <7 years before diagnosis compared with ≥7 years (7.2 vs 3.7 AU; *p* = 0.02), with a trend to higher numbers of anti-CEP-1 antibody positive subjects in the <7 years group (21 % vs 7 %; *p* = 0.06).

**Table 2:** Distribution of antibodies in pre-RA cases stratified by time to RA (dichotomized at median of duration between Ab testing and RA diagnosis, i.e. 7 years). Cut-offs are based on the 98^th^ percentile of controls with the exception of anti-CCP2 antibodies where the manufacturer’s cut-off was applied, and RF where a titre of 1:40 was considered positive. RF; rheumatoid factor. CEP1, cFib and cVim; citrullinated peptides from α-enolase, fibrinogen and vimentin respectively. REP1, Fib and Vim are the arginine-containing control peptides. CPP3 and CPP5 are immunodominant peptides from PPAD, and RPP3 and RPP5 are the arginine-containing control peptides. RgpB; arginine gingipain

Antibodies	Time to diagnosis		
	Time <7 years *n* = 51	Time ≥ 7 years *n* = 52	*p*-value
RF n (%)	15 (29)	9 (18)	0.179
CCP2 n (%)	15 (27)	10 (20)	0.298
CEP1 n (%)	11 (21)	4 (8)	0.056
REP1 n (%)	2 (4)	0 (0)	0.157
cFib n (%)	13 (25)	6 (12)	0.092
Fib n (%)	1 (2)	1 (2)	0.978
cVim n (%)	4 (10)	1 (3)	0.204
Vim n (%)	3 (6)	1 (2)	0.278
CPP3 n (%)	1 (2)	1 (2)	0.989
RPP3 n (%)	3 (6)	2 (4)	0.663
CPP5 n (%)	2 (4)	3 (6)	0.631
RPP5 n (%)	0 (0)	2 (4)	0.149
RgpB n (%)	2 (4)	1 (2)	0.569
RgpB quartiles			
1 (<1280)	25 (48)	26 (51)	0.571
2 (≥1280)	17 (33)	12 (24)	
3 (≥2560)	8 (15)	12 (24)	
4 (≥5120)	2 (4)	1 (2)	
RgpB (u/ml), median (IQR)	1280 (713–1946)	1174 (468–2560)	0.449

### *P.gingivalis* serology

**Fig. 1:**
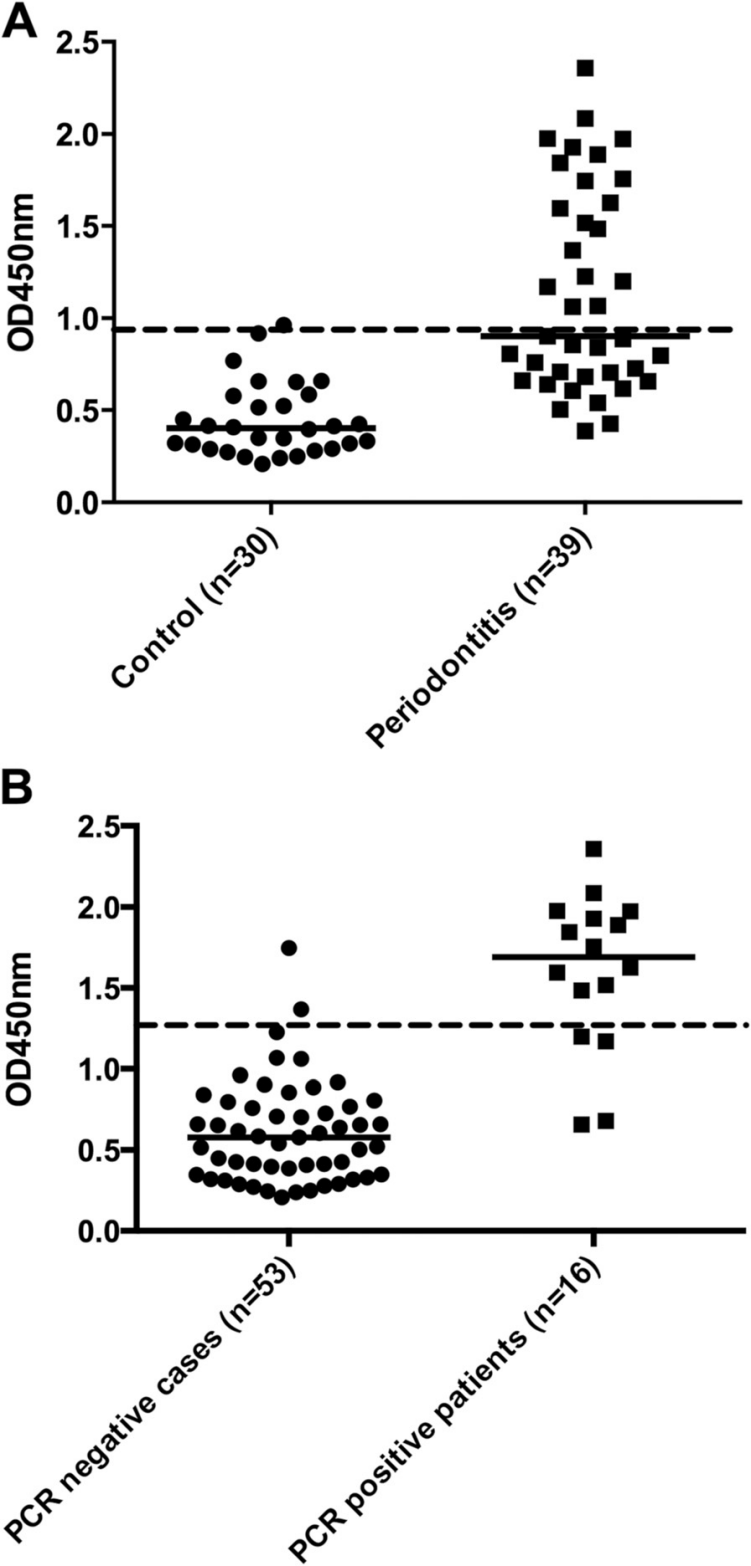
Antibodies to RgpB in (**a**) healthy controls versus patients with periodontitis. Using the 95^th^ percentile of the healthy controls as a cut-off (dashed line), 48 % of patients were positive (*p* < 0.0001); (**b**) healthy and periodontitis subjects that are PCR negative for *P.gingivalis* in dental plaque compared to PCR positive periodontitis patients. In the latter group, 75 % were antibody positive (*p* < 0.0001). Solid lines represent medians

We have previously found anti-RgpB antibody levels to be elevated in subjects with periodontitis [10]. The utility of anti-RgpB antibodies as a marker of infection with *P.gingivalis*, was further tested in a separate cohort of 30 healthy and 39 patients with periodontitis. Using the 95^th^ percentile of the healthy, periodontitis-free controls as a cut-off, 49 % of patients with periodontitis were antibody positive (Fig. 1a). Patients with periodontitis were then divided into two groups based upon the identification of *P.gingivalis* 16S rRNA in subgingival plaque by PCR. Median antibody levels were higher in the PCR-positive patients and, using a higher cut-off based on the 95^th^ percentile of PCR negative patients and controls, 75 % of PCR positive periodontitis patients were also antibody positive compared to 9 % of PCR negative patients (Fig. 1b). This data supports the use of anti-RgpB antibodies as a marker of infection with *P.gingivalis*.

No association with pre-RA was seen with antibodies to RgpB or the two citrullinated PPAD peptides (CPP3 and CPP5). Furthermore, median anti-RgpB antibody levels did not differ between pre-RA subjects that were positive or negative for anti-CCP2 (1300 vs 1149 AU; *p* = 0.36), anti-CEP-1 (1374 vs 1118 AU; *p* = 0.16), anti-cFib (1300 vs 1112 AU; *p* = 0.22), anti-cVim (890 vs 1280 AU; *p* = 0.32) or RF (1139 vs 1280 AU; *p* = 0.77), and these subgroups did not differ from controls. There was also no difference in anti-RgpB antibody levels between subjects known to be RF positive, through testing of pre-RA samples or when this data was recorded in their medical record (‘ever’ RF; *n* = 62), when compared with controls (1184 vs 1280 AU; *p* = 0.81).

### Smoking

The frequency of ever smoking was higher amongst pre-RA cases compared with controls (59 % vs. 47 %, *p* = 0.02). Compared to never smokers, former smokers were nearly 3 times more likely to develop RA (OR 2.95, 95 % CI: 1.40, 6.23; *p* = 0.004), independent of BMI, waist circumference, level of education, physical activity and alcohol intake. No association between current smoking and risk of RA was observed. However, an association was seen for current smokers who were in the lowest intensity bracket (1–15 cigarettes/day: OR 2.15; 95 % CI 1.05–4.37; Table 3). It is conceivable that the lack of association with higher intensity current smoking may reflect a ‘depletion of susceptibles’ i.e. an increased likelihood to develop RA at an earlier age and therefore to not enrol in the EPIC study. We therefore compared both duration and average number of cigarettes smoked. As expected, current smokers smoked for longer than former smokers, but the two groups did not differ in the average number of cigarettes smoked per day during the years of smoking (Table 4). Furthermore, neither the intensity (*p* = 0.64) nor the duration (*p* = 0.57) of smoking were associated with the risk of RA.

**Table 3:** Association of former and current smoking with RA: conditional logistic regression models

	Controls	Pre-RA	Univariate		Multivariate	
			OR (95 % CI)	*p*-value	OR (95 % CI)^a^	*p*-value
Intensity, n (%)						
Model 1						
Never	133 (44)	36 (36)	reference	-	reference	-
Former	59 (19)	28 (28)	1.90 (1.01–3.59)	0.047	2.48 (1.27–4.84)	0.008
Current	80 (26)	30 (30)	1.45 (0.81–2.56)	0.205	1.57 (0.85–2.93)	0.151
Model 2						
Never	133 (43)	36 (35)	reference	-	reference	-
Current, 1–15 cig/day	39 (13)	20 (20)	1.88 (0.98–3.61)	0.059	2.16 (1.06–4.39)	0.034
Current, >16 cig/day	41 (10)	10 (10)	0.95 (0.42–2.13)	0.905	0.97 (0.43–2.28)	0.974
Former, quit ≤10 years	27 (9)	16 (16)	2.18 (1.04–4.55)	0.039	2.79 (1.29–6.07)	0.009
Former, quit >11 years	32 (11)	12 (12)	1.50 (0.66–3.41)	0.328	2.02 (0.84–4.89)	0.115
Current pipe/cigar/occasional	31 (10)	7 (7)	0.93 (0.36–2.41)	0.888	1.24 (0.46–3.34)	0.670

^a^ Models adjusted for BMI, waist circumference, level of education, physical activity index, and alcohol intake

**Table 4:** Antibodies in pre-RA cases stratified by smoking (never/former/current). Cut-offs are based on the 98^th^ percentile of controls with the exception of anti-CCP2 antibodies where the manufacturer’s cut-off was applied, and RF where a titre of 1:40 was considered positive. Ever RF refers to subjects who are known to be RF positive through testing of pre-RA serum or through their post-diagnosis medical records. RF; rheumatoid factor. CEP1, cFib and cVim; citrullinated peptides from α-enolase, fibrinogen and vimentin respectively. REP1, Fib and Vim are the arginine-containing control peptides. CPP3 and CPP5 are immunodominant peptides from PPAD, and RPP3 and RPP5 are the arginine-containing control peptides. RgpB; arginine gingipain

			pre-RA	
	Antibodies	Never smokers	Former smokers	Current smokers
		*n* = 36	*n* = 28	*n* = 37
RF	n (%)	7 (19)	9 (32)	7 (19)
‘Ever’RF	n (%)	20 (56)	15 (54)	26 (70)
CCP2	(u/ml), median (IQR)	1.17 (0–59)	1.74 (0–300)	1.26 (0–300)
	n (%)	7 (19)	11 (39)*	7 (19)
CEP1	(u/ml), median IQR	4.51 (2.22–69)	15.5 (4.20–355)	3.70 (2.99–152)
	n (%)	4 (11)	9 (32)*	2 (5)
REP1	(OD), median (IQR)	0.13 (0.07–0.55)	0.094 (0.05–0.36)	0.076 (0.052–0.27)
	n (%)	2 (6)	0 (0)	0 (0)
cFib	(u/ml), median IQR	2.53 (1.11–77.24)	3.93 (1.30-320)	1.61 (0.94–217)
	n (%)	5 (14)	11 (39)	3 (8)
Fib	(OD), median IQR	0.08 (0.06-0.27)	0.09 (0.06–0.26)	0.08 (0.05–0.25)
	n (%)	1 (3)	0 (0)	1 (3)
cVim	(u/ml), median IQR	14.26 (8.71–114)	29.2 (5.71–401)	16.3 (8.7–240)
	n (%)	1 (4)	2 (8)	2 (8)
Vim	(u/ml), median IQR	8.25 (4.70–26)	8.20 (5.83–24.3)	7.4 (4.6–44.5)
	n (%)	1 (3)	0 (0)	3 (9)
CPP3	(OD), median IQR	0.08 (0.05–0.76)	0.14 (0.08–0.79)	0.10 (0.06–0.33)
	n (%)	1 (3)	1 (4)	0 (0)
RPP3	(OD), median IQR	0.09 (0.06–0.80)	0.13 (0.09–0.94)	0.13 (0.06–0.36)
	n (%)	3 (8)	2 (7)	0 (0)
CPP5	(u/ml), median IQR	26.8 (14.62–71.48)	52.15 (25.66–122)	39.3 (28.3–60.19)
	n (%)	1 (3)	1 (4)	3 (8)
RPP5	(OD), median IQR	27.6 (16.17–35.15)	42.52 (26.8–69.6)	40 (20.99–51.96)
	n (%)	0 (0)	0 (0)	2 (5)
RgpB	(u/ml), median (IQR)	1191 (599–1444)	1555 (966–2560)	948 (553–1976)
	n (%)	1 (3)	2 (7)	0 (0)
	categories, n (%)			
	1 (<1280)	18 (50)	10 (36)	21 (57)
	2 (≥1280)	11 (31)	10 (36)	8 (22)
	3 (≥2560)	6 (17)	6 (21)	8 (22)
	4 (≥5120)	1 (3)	2 (7)	0 (0)
Duration of smoking (years), median (IQR)		-	21 (14–29)	26 (21–34)**
Number of cigarettes/day, median (IQR)		-	9 (6–18)	13 (8–17)
Time since quitting (years), median (IQR)		-	10 (3–16)	-

*p<0.05; **P<0.01

### Smoking and ACPA

**Fig. 2:**
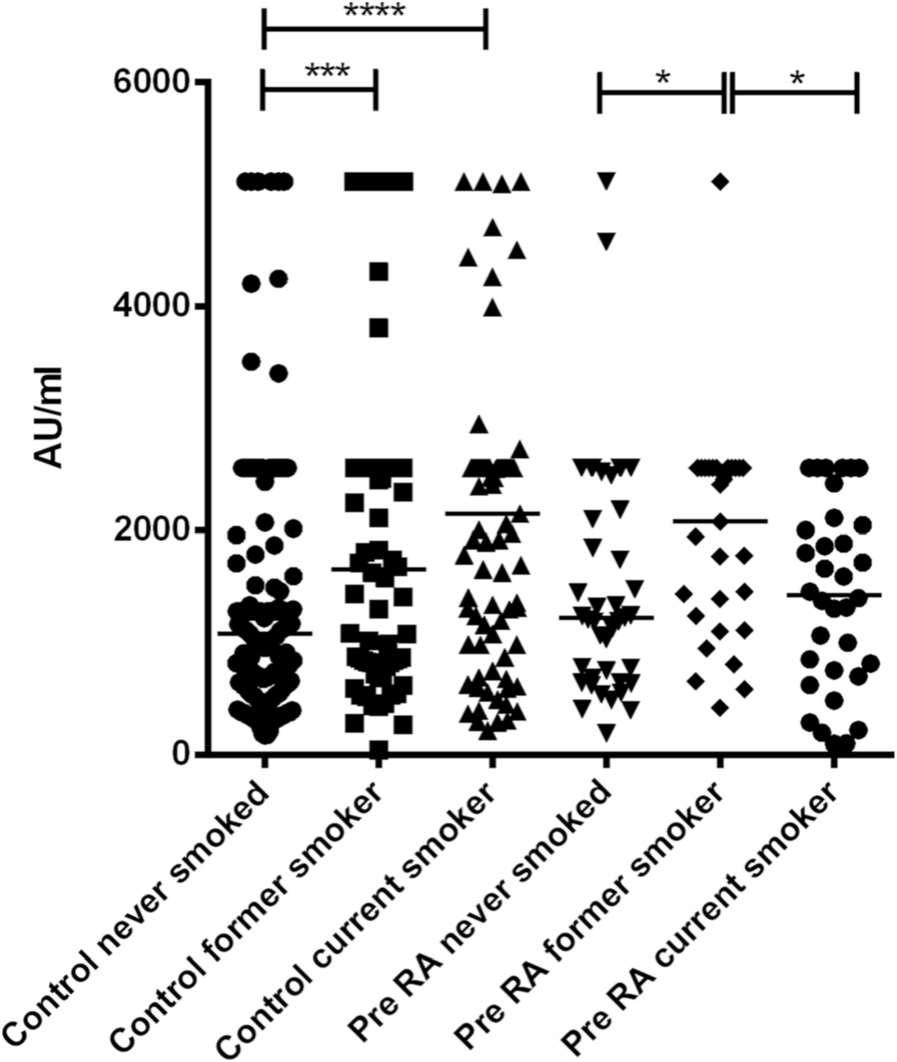
Antibodies to RgpB in pre-RA subjects versus matched controls according to smoking status defined as never, former or current. * *p* < 0.05; *** I < 0.001; **** *p* < 0.0001

Table 4 also shows the relationship between smoking and ACPA positivity amongst the pre-RA cases. Interestingly, a positive association was only observed between former smoking and ACPA. This was confirmed by logistic regression models adjusted for age and sex with statistical significance for anti-CEP-1 and anti-cFib antibodies (data available upon request). Former pre-RA smokers were four times more likely to have anti-CEP-1 (OR 4.06, 95 % CI 1.02, 16.2) and anti-cFib (4.24; 95 % CI 1.2-14.96) antibodies than never pre-RA smokers. In comparison, no association was seen with current smoking [OR 0.54 (CI 0.09-3.73) for anti-CEP-1 and 0.58 (0.13-2.70) for anti-cFib). Interestingly, median antibody levels to both CPP5 and its control peptide, were higher in pre-RA cases who were ever smokers compared to never smokers (CPP5: 47 vs 31 u/ml; *p* = 0.04. RPP5: 42 vs 29 u/ml; *p* = 0.01). In the control group, anti-RgpB levels were highest in current smokers, intermediate in former smokers and lowest in never smokers. In the pre-RA group, antibody levels were highest in the former smoker group (Fig. 2).

## Discussion

In this study of subjects destined to develop RA, we confirmed that smoking is associated with an increased risk of RA. However, whilst smoking was also associated with higher antibody levels to RgpB, a marker of *P.gingivalis* exposure, and to citrullinated peptides from PPAD, these antibodies themselves were not associated with later development of RA or with ACPA.

An association between smoking and RA is well-established, and where observed is usually predominant in the ACPA positive subset [15–17]. This has been less well-studied in the pre-RA phase. Although a large Swedish twin study has recently reported an association of smoking with ACPA positivity in the absence of a diagnosis of RA [27], a number of smaller studies of first-degree relatives (FDRs) have not clearly demonstrated this [28–31]. Recently however, a Swedish study found that ‘ever’ smoking was associated with some but not all ACPA specificities in samples from subjects who later developed RA [18].

An unexpected finding of this study was that former but not current smoking was associated with ACPA and with later development of RA. Whilst this observation may simply be due to the small study size, it is interesting that former and not current smoking was also associated with ACPA. We questioned whether this may reflect earlier development of RA in heavy smokers, who as a result did not enrol in the EPIC study. However, smoking intensity did not differ between former and current smokers, and was not associated with RA. It is also possible that development of ACPA may be associated with intermittent symptoms such as fatigue or arthralgia, or with respiratory disease, which may encourage individuals to cease smoking. However, it is noteworthy that smoking also has anti-inflammatory effects in addition to the more well-known pro-inflammatory ones [32]. These include inhibition of Th1 and Th17 cytokines via stimulation of α7 nicotinic acetylcholine receptors [33], and a reduction in immunoglobulin levels and antibody responses [34–37]. Furthermore, in animal models, nicotine has been demonstrated to reduce the severity of collagen-induced arthritis [38], and smoking to delay its onset and reduce antibody levels to collagen type II and CCP [39]. Therefore another possibility is that smoking creates a suitable mucosal environment for generation of ACPA, but with inhibition of antibody responses until smoking cessation.

Former smoking was associated with a statistically significant risk for antibodies to the CEP-1 and cFib peptides. Other data supports a particularly strong association between anti-CEP-1 and smoking [40–42], and the relationship of anti-CEP-1 antibody levels and positivity with time to diagnosis supports recent suggestions that this antibody specificity may be more closely associated with clinical onset of RA [5].

A number of studies have suggested an association of periodontitis with RA [6]. Periodontal infection is polymicrobial but particular interest has focussed on *P.gingivalis*, in part because it possesses a unique PAD enzyme. Studies in ACPA positive FDRs and in established RA have suggested associations of *P.gingivalis* with RA and ACPA positivity [29, 43–46]. However, a number of these studies employed anti-*P.gingivalis* ELISAs using whole or sonicated bacteria as evidence of past or present infection, and the specificity of such assays is open to question, particularly as the presence of PPAD may generate citrullinated proteins cross-reactive with ACPA. Hitchon et al. did report an association between antibody levels to lipopolysaccharide (LPS) derived from *P.gingivalis*, and ACPA, in patients with RA and their relatives from a North American native population, although the specificity of an assay based on LPS is unclear [29]. We did not find increased antibody levels to RgpB in cases compared with controls. This is similar to our previous findings in established RA, where the median anti-RgpB antibody level did not differ from healthy controls, and was lower than that observed in periodontitis [10]. Mikuls TR et al., whilst finding an association between periodontitis and RA, also did not find an association between antibodies to *P.gingivalis* specific antigens (outer membrane protein and lipopolysaccharide) and RA. Neither did they observe an association with the presence of subgingival *P.gingivalis* [46]. Similarly, whilst there was a high frequency of periodontitis found in patients with new-onset untreated RA, Scher and colleagues found the presence of *P.gingivalis*, as determined by pyrosequencing of subgingival biofilm, to be associated with severity of periodontitis but not with RA. Furthermore, antibodies to *P.gingivalis* specific HtpG P18γ peptides did not differ between cases and controls [47]. Cumulatively these findings suggest that *P.gingivalis* may not account for the association between periodontitis and RA. It is possible that *P.gingivalis* may still have a role in the initiation of RA autoimmunity in a subset of patients and we have previously proposed that immunity to autocitullinated PPAD may be such a trigger [10]. However here, we were not able to demonstrate an antibody response to two different citrullinated PPAD peptides in patients destined to develop RA, suggesting that this is not an early feature in ACPA ontogeny. This is reminiscent of observations made with antibodies to human PAD4 which appear to arise following the ACPA response [48–50]. Importantly, our data suggest that while smoking is associated with both *P.gingivalis* infection and RA, smoking does not increase the risk of RA through its association with *P.gingivalis*.

There are a number of weaknesses in this study. There were varying methods of case ascertainment and the use of hospital discharge databases could bias the cohort towards more severe disease. Due to missing data, not all patients having a physician’s diagnosis of RA could be confirmed as meeting classification criteria. Where such data was available, however, most cases were found to meet 1987 criteria, giving some confidence for those cases where full information was not obtainable. We did not have post-diagnosis samples and we were not able to determine the final ACPA status for most cases. Similarly we cannot rule out P.gingivalis having a specific role at the onset of clinical RA, or related to the severity of RA. Incomplete data also meant that we worked with time to diagnosis rather than time to symptom onset, meaning that some cases labelled as pre-RA may in fact have been prevalent at the time of sampling. However, given that the median length of symptoms prior to diagnosis is 6–8 months, [51, 52] and the median time to diagnosis in our study was 7 years, we do not feel that this should invalidate our findings. Due to the small sample size, we did not analyse gene-environment interactions. Uniquely, this is a southern European pre-RA cohort, and the findings should be replicated in other populations.

## Conclusion

In conclusion, we observed that former smoking, rather than current smoking, was associated with ACPA positivity and incident RA in a cohort of patients destined to develop RA. This effect was independent of an association between smoking and *P.gingivalis* and, if confirmed in other cohorts, suggests a more complex association of smoking with ACPA than previously considered.
