# Erratum to: Smoking, *Porphyromonas gingivalis* and the immune response to citrullinated autoantigens before the clinical onset of rheumatoid arthritis in a Southern European nested case–control study

**DOI:** 10.1186/s12891-016-0916-z

**Published:** 2016-02-05

**Authors:** Benjamin A. Fisher, Alison J. Cartwright, Anne-Marie Quirke, Paola de Pablo, Dora Romaguera, Salvatore Panico, Amalia Mattiello, Diana Gavrila, Carmen Navarro, Carlotta Sacerdote, Paolo Vineis, Rosario Tumino, David F. Lappin, Danae Apatzidou, Shauna Culshaw, Jan Potempa, Dominique S. Michaud, Elio Riboli, Patrick J. Venables

**Affiliations:** Rheumatology Research Group, Centre for Translational Inflammation Research, Queen Elizabeth Hospital Birmingham, Birmingham, B15 2WB UK; Kennedy Institute of Rheumatology, University of Oxford, Oxford, UK; School of Public Health, Imperial College London, London, UK; CIBER-OBN (Fisiopatología de la Obesidad y Nutrición), Madrid, Spain; Department of Clinical and Experimental Medicine, Federico II University of Naples, Naples, Italy; Department of Epidemiology, Murcia Regional Health Council, Murcia, Spain; CIBER Epidemiología y Salud Pública (CIBERESP), Murcia, Spain; Human Genetics Foundation, Turin, Italy; Cancer Registry and Histopathology Unit, “Civic - M.P.Arezzo” Hospital, ASP Ragusa, Ragusa, Italy; University of Glasgow Dental School, University of Glasgow, Glasgow, UK; Faculty of Biochemistry, Biophysics and Biotechnology, Jagiellonian University, Krakow, Poland; Oral Health and Systemic Research Group, School of Dentistry, University of Louisville, Louisville, USA; Department of Epidemiology, Brown University School of Public Health, Providence, USA

## Erratum

After publication of the original article [1], the publisher was notified that there was a spelling error in one of the author’s surnames. Danae Apatzidou’s surname was erroneously spelled ‘Apazidou’.
